# Social Support as Technostress Inhibitor: Even More Important During the COVID-19 Pandemic?

**DOI:** 10.1007/s12599-023-00799-7

**Published:** 2023-03-21

**Authors:** Julia Lanzl

**Affiliations:** 1 Branch Business & Information Systems Engineering of the Fraunhofer FIT, Augsburg, Germany; 2 Chair of Digital Management, University of Hohenheim, Stuttgart, Germany; 3 Research Center Finance and Information Management, Augsburg, Germany

**Keywords:** Technostress, Technostress inhibitors, Social support, Longitudinal data, Structural equation modeling

## Abstract

Due to ongoing digitalization and the social distancing measures that came along with the COVID-19 pandemic, the working conditions and environments have changed for many individuals. Because of increased telework, the use of digital technologies for communicating and collaborating at work has been intensified, which can cause technostress. With longitudinal data from two surveys – one before and one during the COVID-19 pandemic – the paper analyzes the relationship between four social support dimensions (supervisor support, co-worker support, sense of community at work, and family support) and technostress creators. The study shows that social support can be an effective inhibitor of technostress creators. However, social support dimensions have to be differentiated in that regard. Further, the results show that the inhibiting effect of family support has become even more important during the COVID-19 pandemic. The results contribute to technostress research and research with regard to the new normal of working after the pandemic.

**Supplementary Information:**

The online version contains supplementary material available at 10.1007/s12599-023-00799-7.

## Introduction

The COVID-19 pandemic has changed the working conditions for many people. Due to the social distancing measures for fighting the pandemic, many employees were asked to engage in telework and work from home. In July 2020, one third of the employees in the European Union worked entirely from home, almost 50% at least partly (Ahrendt et al. 2020). Similar numbers were reached in the United States with up to 37% of people working from home full-time in April 2020 – an increase of 32 percentage points compared to the pre-pandemic time (Yang et al. 2022). These developments have led to changed expectations of employees since they demand more flexibility even after the pandemic-caused restrictions (Mercer 2021). However, the extended work from home also came with a higher amount of digital work and less contact with co-workers, but also with increased contact with family members since many were working from home together.

One phenomenon that goes along with digital work is technostress, which refers to “stress that users experience as a result of their use of IS in the organizational context” (Tarafdar et al. 2015, p. 103). Technostress is associated with lower job satisfaction, lower productivity, and a higher risk of burnout (Tu et al. 2008; Ragu-Nathan et al. 2008; e.g., Day et al. 2012). To address such negative outcomes, literature on technostress has investigated potential mitigation strategies. While coping literature deals with behavioral, cognitive, and perceptional efforts of individuals (Weinert et al. 2020), literature on technostress inhibitors focuses on organizational or environmental mechanisms that reduce technostress creators or its negative consequences (Ragu-Nathan et al. 2008). Such inhibitors are, for example, the fostering of learning to deal with digital technologies, the provision of technical support, or the involvement of employees when launching new digital technologies (Ragu-Nathan et al. 2008).

However, the COVID-19 pandemic not only forced employees to telework or work from home, it also changed companies in general, as they had to adapt different parts of their organizations to the new situation (Richter 2020). This had the consequence that organizational measures, which usually would help employees in mitigating technostress, were less available, for example, for the following reason: Many companies introduced communication and collaboration tools almost over night in order to be able to stay connected. This extremely fast introduction of new digital technologies made it harder for organizations to engage in adequate change management and to involve employees in the choice of the new digital technologies or provide extensive training before the change. Such organizational measures have been found to inhibit technostress; however, they were less available during the COVID-19 pandemic.

The overarching goal of this paper is to understand whether there is an alternative source of support for mitigating technostress when organizational measures are less available. Results from Ahrendt et al. (2020) suggest that this source of support could be found in the social environment, as they discovered that the perception that employees receive help and social support from supervisors or co-workers whenever needed did not change despite the increased digital work during the COVID-19 pandemic. Such social support has already been considered as inhibitor of workplace stress in psychology research (e.g., Barrera 1986; Eisenberger et al. 2002; Sass et al. 2011). There are different sources of social support (e.g., supervisors, co-workers, family members) (Barrera 1986), and since different dimensions of social support have been shown to be beneficial for mitigating work stress in psychology research, we aim to understand whether social support can inhibit technostress as well. The second goal of our research is to analyze whether the importance of social support is different during times of intense telework (i.e., during the COVID-19 pandemic) when organizational support may be less available. Therefore, we pose the following research questions:

*RQ1: Are different dimensions of social support effective technostress inhibitors?*

*RQ2: Was social support as technostress inhibitor more important during the COVID-19 pandemic?*

To answer these questions, we draw on literature from psychology on the effect of social support on work stress creators and outcomes and develop hypotheses about the association of different social support dimensions (supervisor support, co-worker support, sense of community at work, and family support) with technostress creators (techno-invasion, techno-overload, techno-complexity, techno-insecurity, and techno-uncertainty) as well as with the changes in times of telework. We collect longitudinal empirical data on the constructs at two points of time (i.e., before and during the COVID-19 pandemic) and analyze the data by structural equation modeling and regression analysis with interaction effects.

The paper is structured as follows: Sect. 2 introduces the theoretical background on technostress literature, technostress inhibitors, and other stress mitigation constructs from psychology literature. Section 3 develops the hypotheses. Section 4 describes the study design and procedures, and Sect. 5 displays the corresponding results. Section 6 discusses the results as well as the theoretical contribution and practical implications of the findings. Finally, Sect. 7 concludes the paper.

## Theoretical Background

### Technostress and Technostress Creators

Studies on technostress can be traced back to the clinical psychologist Brod (1982), who coined the term and described the phenomenon as an individual’s inability to deal with new technology in a healthy way, which leads to a stressful experience. In psychology literature, Lazarus and Folkman (1984, p. 19) defined stress in their transactional model of stress as a “particular relationship between the person and the environment that is appraised by the person as taxing or exceeding his or her resources and endangering his or her well-being.” For technostress, the demands result from the use of digital technologies.

Tarafdar et al. (2007) and Ragu-Nathan et al. (2008) have defined five technostress creators, which, to date, are the most established and researched ones in IS literature: techno-invasion, techno-overload, techno-complexity, techno-insecurity, and techno-uncertainty. Table 1 shows the definitions of the five technostress creators.

**Table 1:** Definitions of technostress creators

Technostress creator	Definition	Source
Techno-invasion	“Techno-invasion describes the invasive effect of [digital technologies] in situations where employees can be reached anytime and feel the need to be constantly connected, thus blurring work-related and personal contexts.”	Ragu-Nathan et al. 2008, p. 427
Techno-overload	“Techno-overload describes situations where [digital technologies] force users to work faster and longer.”	Ragu-Nathan et al. 2008, p. 427
Techno-omplexity	“Techno-complexity describes situations where the complexity associated with [digital technologies] leads users to feel inadequate with regard to their computer skills and forces them to spend time and effort in learning and understanding [digital technologies].”	Ragu-Nathan et al. 2008, p. 427
Techno-uncertainty	“Techno-uncertainty refers to contexts where continuing [digital technology] changes and upgrades unsettle users and create uncertainty so that they must constantly learn and educate themselves about new [digital technologies].”	Ragu-Nathan et al. 2008, p. 427
Techno-insecurity	“Techno-insecurity is associated with situations where users feel threatened about losing their jobs, either because of automation from [digital technologies] or to other people who have a better understanding of [digital technologies].”	Ragu-Nathan et al. 2008, p. 427

Technostress can be either framed as negative (also called techno-distress; i.e., digital technologies are appraised as a threat) or as positive (also called techno-eustress; i.e., digital technologies are appraised as challenging or thrilling) (Tarafdar et al. 2019). Similarly, some existing studies differentiated hindrance and challenge technostress (e.g., Benlian 2020; Califf et al. 2020). However, in the scope of this paper, we only regard the negative side of technostress and the aim to minimize it.

Most technostress research focuses on the working environment with few exceptions looking into the private use of digital technologies and related technostress, such as Salo et al. (2022). Further, some research exists considering the boundaries between work and private lives. Literature suggests that there can be both positive and negative spill-over effects in both directions (e.g., Grzywacz and Marks 2000). For example, when there is a disagreement between the individual and the spouse this may negatively affect the individual in its work domain and vice versa. Considering these spill-over effects is especially important when employees work remotely, as observed during the COVID-19 pandemic, because work and private lives take place at the same location (Oksanen et al. 2021). Benlian (2020) considered spill-over effects between both domains of life and investigated the effects of technostress creators on private outcomes. He found positive as well as negative spill-overs on satisfaction with the individual’s partnership: hindrance technostress creators were negatively associated with partnership satisfaction (via negative affect) and challenge technostress creators were positively associated with partnership satisfaction (via positive affect). However, his study took place before the COVID-19 pandemic when telework and work from home was much less frequent, and it did not consider spill-over effects in the other direction (i.e., from the private domain to the work domain). Thus, we aim to add to his research in the times of the COVID-19 pandemic and investigate whether there can be a positive spill-over effect of the private domain to experiencing technostress creators in the work domain.

### Organizational Measures to Mitigate Technostress

Since technostress has been found to have negative effects on individuals and organizations such as reduced job satisfaction, increased burnout, or lower organizational commitment (Tarafdar et al. 2007; e.g., Day et al. 2012; Ragu-Nathan et al. 2008), much research focuses on the mitigation of technostress. Thereby, literature can be divided into two streams: technostress inhibitors, and coping. Technostress inhibitors refer to “organizational mechanisms that have the potential to reduce the effects of technostress” (Ragu-Nathan et al. 2008, p. 422). Coping, in contrast, focuses on the individual perspective and “investigates how users themselves aim to reduce technostress by deploying behavioral, cognitive, and perceptional efforts” (Weinert et al. 2020, p. 1203). In our study, we focus on mechanisms from the individual’s environment and, thus, draw on literature of technostress inhibitors which also are available in the individual’s environment.

Several studies can be found that investigate the effects of technostress inhibitors as organizational measures to mitigate technostress (e.g., Ragu-Nathan et al. 2008; Tarafdar et al. 2010; e.g., Day et al. 2012). As Sarabadani et al. (2018) summarized, some studies investigated the effect of technostress inhibitors on technostress creators (e.g., Tarafdar et al. 2010, 2011, 2015), some focused on the direct effect on technostress outcomes (e.g., Day et al. 2012; Ragu-Nathan et al. 2008; Tarafdar et al. 2010, 2011), and some analyzed the moderating effect on the relationship of technostress creators and outcomes (e.g., Ahmad et al. 2014; Ragu-Nathan et al. 2008; Tu et al. 2008).

The most studied technostress inhibitors are three organizational mechanisms: literacy facilitation (i.e., promoting the sharing of knowledge about digital technologies), involvement facilitation (i.e., involving employees in the change process when introducing new digital technologies), and technical support provision (i.e., the provision of an adequate end-user support for problems with digital technologies). Tarafdar et al. (2015), for example, found the three to be negatively associated with technostress creators. Direct negative effects on technostress creators have also been found by Tarafdar et al. (2010) (for involvement facilitation) and Tarafdar et al. (2011) (for involvement facilitation, technical support provision, and innovation support). For the direct effects on technostress outcomes, the three inhibitors and other inhibitors such as innovation support, stress management trainings, and job control have been found to have a positive effect on, for example, end-user satisfaction, job satisfaction, organizational commitment, continuance commitment, and productivity (Ahmad et al. 2014; Fuglseth and Sørebø 2014; Ragu-Nathan et al. 2008; Tarafdar et al. 2010, 2011; Tu et al. 2008), and a negative effect on burnout (Day et al. 2012). Regarding the moderating effect of technostress inhibitors on the relationship between technostress creators and outcomes, Ahmad et al. (2014), for example, found technical support to moderate the relationship between techno-overload and organizational commitment. Other studies such as Ragu-Nathan et al. (2008), Tu et al. (2008), and Hung et al. (2011) did not find moderating effects of technostress inhibitors.

To summarize these findings, it can be stated that technostress inhibitors can indeed be positive in mitigating technostress or its negative outcomes. However, results differ in which phase in the technostress process the inhibitors have been shown to mitigate technostress (either at the beginning where technostress creators meet the individual or after the appraisal of technostress when it comes to negative outcomes of technostress). Also in the prior studies, different technostress creators have been investigated. However, mostly they are operationalized as one higher-order technostress construct (e.g., Ragu-Nathan et al. 2008; Tarafdar et al. 2011). But because of the high complexity of the technostress process, it might be better to differentiate the technostress creators on a first-order level to carve out possible differences in their relationship to technostress inhibitors. Therefore, we will consider the five technostress creators (techno-invasion, techno-overload, techno-complexity, techno-insecurity, and techno-uncertainty) individually.

### Social Support as Measure to Mitigate (Techno) Stress

Another category of stress mitigation measures can be found in psychology literature. There, researchers have investigated different dimensions of social support and their relationship with different types of stress and strain (Barrera 1986). This social support is not an organizational measure as the previously described technostress inhibitors, but rather a soft mechanism between different individuals in organizations. One important dimension of social support is perceived social support, which refers to the “perceived availability and adequacy of supportive ties” (Barrera 1986, p. 416). Another dimension of social support is social embeddedness, which “refers to the connections that individuals have to significant others in their social environments” (Barrera 1986, p. 415).

There are many studies that investigated perceived social support and social embeddedness and their relationship to stress in the organizational context. Witt and Carlson (2006, p. 347), for example, investigated perceived organizational support and define it as “the employee’s assessment of the extent to which the organization is ‘on my side.’” Organizational support (i.e., social support from various sources in the organization) has been found to be associated with increased satisfaction, job performance, and continuance commitment (Eisenberger et al. 1990; Patrick and Laschinger 2006). More specific than organizational support in general, support for individuals at their workplaces can stem from different groups of people of an individual’s environment: supervisors, colleagues, and family members (Sass et al. 2011; Wolgast and Fischer 2017; e.g., Mansour and Tremblay 2016).

One technostress paper has so far also applied psychology literature on social support to their study. Weinert et al. (2020) investigated whether instrumental or emotional backing can reduce the strain response of the technostress creator techno-unreliability (i.e., the experience of malfunctions and other hassles with digital technologies) in an experimental study where participants were confronted with a computer freeze. In their study, they conceptualized social support as a coping strategy that individuals pursue in the situation itself after being confronted with the malfunction. The authors found that social support can be an effective coping strategy, but they did not investigate the relationship of social support to the technostress creator itself.

According to Barrera (1986), social support can relate to stress and stress outcomes in different ways: by directly affecting the occurrence of stress events, perceived stress, or stress outcomes. This is along the lines with prior literature on technostress inhibitors (Sarabadani et al. 2018). With Weinert et al.’s (2020) study being the only existing technostress study that has considered social support as coping strategy, technostress literature has neglected dimensions of social support and their possible consideration as technostress inhibitors (in contrast to being coping strategies), which thus would directly affect technostress creators. To close this gap, we aim at transferring knowledge about different dimensions of social support from psychology literature to the context of technostress and analyze their effect on technostress creators. Thereby, we especially aim to understand whether the importance of four dimensions of social support (i.e., supervisor support, co-worker support, sense of community at work, and family support) have changed in times of intense mobile and telework (i.e., during the COVID-19 pandemic).

## Hypotheses Development

With our study, we aim to understand whether social support can be effective in mitigating technostress. According to Lazarus and Folkman (1984), stress is the result of an interplay of environmental demands and the individual’s resources. This is in line with related psychology theories like the conservation of resources theory (Hobfoll 1989) or the job demands-resources model (Demerouti et al. 2001). In all these theoretical bases, social support has been considered as one important resource for inhibiting stress creators (Barrera 1986). Thus, more precisely, we aim to understand whether social support shows the same effect as a resource for inhibiting technostress creators than it has shown for other stress creators.

As social support is a multidimensional construct (Barrera 1986), we want to understand the relationship of different dimensions of social support with technostress creators. Therefore, we investigate four dimensions of social support: supervisor support, co-worker support, sense of community at work, and family support. The first two and the last of them refer to the “perceived social support” dimension and the third one refers to the “social embeddedness” dimension of Barrera (1986). Table 2 displays the definitions of the four dimensions of social support.

**Table 2:** Definitions of social support dimensions

Dimension of social support	Definition	Source
Supervisor support	Supervisor support is the “degree to which supervisors value their [employees’] contributions and care about their well-being.”	Eisenberger et al. (2002), p. 565
Co-worker support	Co-worker support refers to a “cooperative peer-level effort amongst employees to provide work-related assistance.”	Jia et al. (2008), p. 307
Sense of community at work	Sense of community refers to “the overall quality of social interaction at work.”	Leiter and Maslach (2003), p. 98
Family support	Family support is defined as the “degree of […] support [from family members] employees perceive as directed at their roles as worker.”	King et al. (1995), p. 236

The four dimensions of social support have been found to have different effects on stress creators or its outcomes. For supervisor support, Sass et al. (2011) found it to be negatively associated with workload stressors and job dissatisfaction. Sosik and Godshalk’s (2000) results show less job stress of employees when their leaders engage in a mentoring function. Regarding co-worker support, Sass et al. (2011) as well as Wolgast and Fischer (2017) detected negative effects on job dissatisfaction and strain. McCarty et al. (2007) discovered a negative effect of camaraderie on work-related strain. For family support, Barnett et al. (2012) as well as Mansour and Tremblay (2016) found it to be negatively associated with job strain and Asbari et al. (2021) found a positive effect on job satisfaction. Lastly, regarding sense of community at work, Cicognani et al. (2009) found a negative correlation between sense of community and burnout. In the same regard, Gascón et al. (2021) detected negative effects on burnout. They also found sense of community to negatively moderate the relationship between workload and cynicism and lack of job fulfillment (Gascón et al. 2021).

In line with these findings, we propose a negative relationship of social support (i.e., supervisor support, co-worker support, sense of community at work, and family support) with technostress creators. The reasons are as follows: Higher social support gives employees the feeling that they can expect help when problems with digital technologies occur and, thus, feel less threatened in the first place. For supervisor support, for example, it is easier for an employee to talk to their supervisors about their fear of losing the job (i.e., techno-insecurity) if the supervisor is concerned with the employee’s needs. Also, co-workers may be expected to help when individuals experience changes or updates to their digital technologies (i.e., techno-uncertainty) since these changes also affect the co-workers. Thus, the feeling of being in the same boat regarding these demands of the changed digital technologies may help to not perceive this technostress creator as high or hindering. To sum it up, we pose the following hypothesis:

### H1

Social support dimensions a) supervisor support, b) co-worker support, c) sense of community at work, and d) family support) are negatively related to technostress creators.

The COVID-19 pandemic was a sudden shock for the whole world in general and the working environments in particular. Psychology literature suggests that in such situations of crises, social support can be effective to reduce negative outcomes and promote positive adaptation to the new situation (Saltzman et al. 2020). During the COVID-19 pandemic, many employees were forced to telework and work from home. Thus, their working environment and the availability of organizational resources changed rapidly. But this was also the fact for the employees that still worked in their organization’s office. Whereas, before the pandemic, many employees worked in the organization’s office and were surrounded by their co-workers and supervisors, individuals now worked from home or in much less frequented offices. Since many organizations were not used to communicating and collaborating remotely, new digital tools such as Zoom or Microsoft Teams were introduced almost over night to be able to stay in contact with co-workers and supervisors (Ben-Zvi and Luftman 2021). Oksanen et al. (2021), for example, found that social media communication increased during the pandemic in all occupational fields and, as a result, technostress increased as well. These rapid introductions of new digital technologies made extensive change management impossible and organizations did not find much time to consider or experiment with alternative digital technologies or to train their employees to use the newly introduced digital technologies (Carroll and Conboy 2020). Thus, the traditional technostress inhibitors such as involvement facilitation, literacy facilitation, and technology support provision were less available. Therefore, not only demands changed but employees had to adapt to the new environmental conditions and find effective and available sources of support. Thus, we hypothesize:

### H2

The negative relationship between social support and technostress creators is stronger during the COVID-19 pandemic.

## Study Design and Procedures

To test our hypotheses empirically, we conducted a longitudinal online survey and measured all constructs from the research model in the questionnaire at two points of time with the same participants (within-subject design). At the beginning of the questionnaire, participants were informed about the purpose of the study and introduced to the topic. The questionnaire itself contained questions on technostress creators, social support, and demographics in order to answer the research questions. Since the study was part of a larger research project, it included further questions that are not presented in this paper.

For technostress creators, we used the items from Ragu-Nathan et al. (2008). For supervisor support and family support, we built on the scale by Graen and Uhl-Bien (1995) and changed the style of the questions into statements (e.g., “How well does your leader understand your job problems and needs?” into “My leader understands my job problems and needs.”) to match the style of the other items in the survey. Items for co-worker support and sense of community at work were collected from Burr et al. (2019). Where possible, we used validated German translations of the items. For all other items, we translated the English versions to German. Appendix A (available online via http://link.springer.com) provides an overview of all items.

We recruited participants via a German panel provider and paid a small compensation for their participation. The first survey was conducted in March 2019 (T1). In December 2020, during the second lockdown in Germany, we surveyed the same participants for the second time (T2). At that time, Germany was confronted with the second COVID-19 wave. There were strict contact restrictions (only five people from two households were allowed to meet) and restaurants, retail stores, museums, etc. were closed. Working from home was not yet mandatory by law (not until January 2021) but recommended by many experts, and many employers followed these recommendations.

In the first survey in T1, 5005 complete answers could be obtained. These participants were chosen randomly by the panel provider. However, the sample was chosen to mostly represent the German working population regarding age, gender, and state. As the study was part of a large research project which had a focus on knowledge workers, we included a higher proportion of knowledge workers (approximately two thirds) than the German workforce overall contains, but apart from this focus the participants were chosen randomly regarding, for example, their job, position, industry, or educational background. All in all, we have a diverse sample regarding different criteria, but we cannot assume it to be representative of the German or global workforce. In T2, 637 individuals of the initial 5005 participants again responded to the invitation to the survey and had not changed their employer or experienced any major changes in their work settings since the first survey.

Of the 637 final respondents, 41.1% were female and 58.9% were male. On average, respondents were 47 years old at the first time of participation. Table 3 displays further demographics of the sample.

**Table 3:** Demographics of the sample

	Absolute frequency	Relative frequency (%)
*Gender*		
Female	375	58.9
Male	262	41.1
*Age in years*		
Below 25	5	0.8
25–34	77	12.1
35–44	174	27.3
45–54	200	31.4
55–64	177	27.8
65 and above	4	0.6
*Education*		
Primary/Secondary degree	117	18.4
Apprenticeship	219	34.4
University degree/University of applied science degree	270	42.4
Doctorate	31	4.9
*Working hours per week*		
Below 20	14	2.2
20–34	81	12.7
35–40	485	76.1
Above 40	57	8.9
*Marital status*		
Single	149	23.4
Separated/Divorced	44	6.9
In a relationship	93	14.6
Married/Civil union	340	53.4
Widowed	11	1.7
*Persons under 18 in household*		
0	460	72.2
1	82	12.9
2 or more	95	14.9
*Persons over 18 in household (Excluding oneself)*		
0	189	29.7
1	377	59.2
2 or more	71	11.1
*Elderly people in household with care work commitments to oneself*		
Yes	15	2.4
No	622	97.6
*Industry*		
Financial and insurance service providers	68	10.7
Corporate service provider	60	9.4
Public and other private service providers	227	35.7
Information and communication	67	10.5
Real estate and housing	15	2.4
Trade, transport, and hospitality	79	12.4
Construction industry	29	4.6
Manufacturing industry (excluding construction)	88	13.8
Agriculture and forestry, fisheries	3	0.5

## Results

Our analysis strategy was threefold: First, we conducted paired t-tests in order to compare the variables at the two points in time (T1 and T2). Second, we assessed two structural equation models at the two points of time by means of covariance-based structural equation modeling (CB-SEM). Each of the models consisted of the five technostress creators as dependent variables and the four social support dimensions as independent variables. Each technostress creator was explained by each social support dimension. We started with an evaluation of the measurement models and proceeded by assessing the structural models and testing our first hypothesis. Third and last, we conducted clustered regression analyses to test whether changes in paths between the two points of time were significant and to test our second hypothesis.

### Comparison of Variables for T1 and T2

We started with a mean comparison of our variables at both points of time and conducted paired t-tests to test whether mean differences were statistically significant. Table 4 shows the results. Only techno-invasion, techno-uncertainty, and family support showed significant differences between T1 and T2. Techno-invasion had become slightly higher during the COVID-19 pandemic, whereas techno-uncertainty and family support had decreased. The other technostress creators and social support dimensions did not change significantly.

**Table 4:** Results of paired *t*-tests

Construct	Mean T1	Mean T2	Difference (T2 – T1)	*p*-value of paired *t*-test		*f*^2^
Techno-invasion^1^	0.902	1.021	0.119	0.002	**	0.119
Techno-overload^1^	1.429	1.389	− 0.040	0.333		− 0.037
Techno − complexity^1^	1.063	1.130	0.067	0.077		0.067
Techno − insecurity^1^	1.726	1.467	− 0.258	0.000	***	− 0.251
Techno − uncertainty^1^	1.042	0.977	− 0.066	0.059		− 0.068
Supervisor support^1^	2.433	2.390	− 0.042	0.145		− 0.042
Co-worker support^2^	2.427	2.464	0.037	0.261		0.043
Sense of community^2^	2.925	2.948	0.023	0.469		0.028
Family support^1^	2.838	2.728	− 0.110	0.001	***	− 0.125

***p < 0.001, **p < 0.01, *p < 0.05

^1^Scale is ranging from 0 to 4

^2^Scale is ranging from 0 to 5

### Assessment of the Measurement Models at T1 and T2

Next, we used CB-SEM to assess the two models at T1 and T2 and started with an evaluation of the measurement models. For the reliability assessment, we used Cronbach’s Alpha. All scales’ values for Cronbach’s Alpha exceeded the threshold of 0.708 with a minimum of 0.810, which indicates internal consistency reliability (Nunnally and Bernstein 1994). Also, convergent validity was satisfactory as the minimum of all indicators’ outer loadings was 0.623 and the minimum average variance extracted (AVE) was 0.581. For discriminant validity, we examined whether each construct’s square root of the AVE was higher than the highest correlation with other constructs (Fornell-Larcker criterion). The data met this criterion. Thus, discriminant validity was supported for both models. Table 5 and Table 6 show means, standard deviations (SD), loadings, Cronbach’s Alpha (Alpha) values as well as the AVE values for all constructs at T1 and T2. Information on the Fornell-Larcker criterion can be found in Appendix B.

**Table 5:** Descriptive statistics, main factor loadings, Cronbach’s Alpha, and AVE at T1

Construct	# Items	Mean	SD	Loadings	Alpha	AVE
Techno-invasion^1^	3	0.902	1.213	0.633–0.891	0.815	0.612
Techno–overload^1^	4	1.429	1.305	0.710–0.892	0.896	0.693
Techno-complexity^1^	5	1.063	1.166	0.770–0.883	0.912	0.680
Techno-insecurity^1^	5	1.726	1.197	0.694–0.825	0.871	0.581
Techno-uncertainty^1^	5	1.042	1.238	0.756–0.875	0.875	0.639
Supervisor support^1^	6	2.433	1.186	0.720–0.899	0.933	0.706
Co-worker support^2^	4	2.499	1.186	0.800–0.852	0.810	0.681
Sense of community^2^	2	2.925	0.844	0.901–0.909	0.901	0.820
Family support^1^	5	2.838	1.059	0.623–0.882	0.879	0.604

^1^Scale is ranging from 0 to 4

^2^Scale is ranging from 0 to 5

**Table 6:** Descriptive statistics, main factor loadings, Cronbach’s Alpha, and AVE at T2

Construct	# Items	Mean	SD	Loadings	Alpha	AVE
Techno-invasion^1^	3	1.021	1.214	0.659–0.870	0.813	0.605
Techno-overload^1^	4	1.389	1.252	0.771–0.893	0.915	0.729
Techno-complexity^1^	5	1.130	1.169	0.755–0.886	0.922	0.705
Techno-insecurity^1^	5	0.977	1.134	0.709–0.850	0.887	0.618
Techno-uncertainty^1^	5	1.467	1.205	0.783–0.921	0.906	0.717
Supervisor support^1^	6	2.390	1.194	0.761–0.904	0.939	0.726
Co-worker support^2^	4	2.521	1.003	0.810–0.887	0.836	0.720
Sense of community^2^	2	2.948	0.838	0.909–0.915	0.908	0.832
Family support^1^	5	2.728	1.073	0.758–0.886	0.901	0.647

^1^Scale is ranging from 0 to 4

^2^Scale is ranging from 0 to 5

### Assessment of Structural Models at T1 and T2

We proceeded with the assessment of the structural models. Table 7 displays several fit indices that we used to assess the models’ fit. Almost all indices comply with the respective thresholds indicating satisfactory model fit for both models.

**Table 7:** Fit indices for the research models at T1 and T2

Fit measures		Threshold	Source of threshold	Model T1	Model T2
Global measures	RMSEA	< 0.06	Lei and Wu (2007)	0.054✓	0.061Χ
	SRMR	< 0.05	Gefen et al. (2000)	0.049✓	0.050✓
Incremental measures	NFI	> 0.90	Gefen et al. (2000)	0.903✓	0.900✓
	TLI	> 0.90	Gefen et al. (2000)	0.927✓	0.918✓
	CFI	> 0.90	Gefen et al. (2000)	0.936✓	0.927✓
Parsimony	AGFI	> 0.80	Gefen et al. (2000)	0.835✓	0.802✓

Root mean square error of approximation (RMSEA), standardized root mean square residual (SRMR), normed fit index (NFI), tucker-lewis index (TLI), comparative fit index (CFI), adjusted goodness of fit index (AGFI)

✓indicates that a threshold is met, Χ indicates that it is not met

After the evaluation of the models’ fit, we tested our hypothesis about the relationship of social support with technostress creators. Table 8 presents the path estimates for both models as well as their significance level and the effect size (*f*^2^).

**Table 8:** Results of structural models

Relationship	Model T1				Model T2			
	Estimate	*p*-value	Sig	*f*^2^	Estimate	*p*-value	Sig	*f*^2^
Supervisor support → Techno-invasion	0.117	0.012	*	0.018	0.112	0.037	*	0.005
Supervisor support → Techno-overload	− 0.035	0.449		0.009	− 0.054	0.301		− 0.004
Supervisor support → Techno-complexity	− 0.007	0.884		0.004	− 0.018	0.719		− 0.002
Supervisor Support → Techno-insecurity	0.107	0.025	*	0.012	− 0.029	0.576		− 0.003
Supervisor support → Techno-uncertainty	0.121	0.014	*	0.010	− 0.026	0.624		0.000
Co-Worker support → Techno-invasion	− 0.100	0.082		0.008	0.101	0.128		0.002
Co-worker support → Techno-overload	− 0.044	0.444		0.003	0.122	0.058		0.004
Co-worker support → Techno-complexity	0.006	0.923		− 0.001	0.092	0.150		0.003
Co-worker support → Techno-insecurity	− 0.114	0.053		0.010	0.172	0.007	**	0.209
Co-worker support → Techno-uncertainty	− 0.060	0.318		0.004	0.126	0.060		− 0.157
Sense of community at work → Techno-invasion	− 0.374	0.000	***	0.113	− 0.303	0.000	***	0.053
Sense of community at work → Techno-overload	− 0.276	0.000	***	0.057	− 0.320	0.000	***	0.058
Sense of community at work → Techno-complexity	− 0.335	0.000	***	0.087	− 0.290	0.000	***	0.051
Sense of community at Work → Techno-insecurity	− 0.291	0.000	***	0.063	− 0.434	0.000	***	0.125
Sense of community at work → Techno-uncertainty	− 0.113	0.050		0.008	− 0.138	0.026	*	0.010
Family support → Techno-invasion	0.062	0.190		0.004	− 0.229	0.000	***	0.043
Family support → Techno-overload	0.030	0.527		0.001	− 0.123	0.009	**	0.007
Family support → Techno-complexity	− 0.032	0.492		0.001	− 0.174	0.000	***	0.025
Family support → Techno-insecurity	0.012	0.808		0.000	− 0.154	0.001	***	0.019
Family support → Techno-uncertainty	0.093	0.061		0.008	− 0.092	0.059		0.006

*p <  0.05, **p < 0.01, ***p < 0.001,

The results show differences between the social support dimensions. Supervisor support is significantly related to techno-invasion, techno-insecurity, and techno-uncertainty in T1 and with techno-invasion in T2. However, the relationship is positive and not negative as expected. For co-worker support, we only find one significant relation with techno-insecurity in T2. Again, it is positive and thus other than hypothesized. Sense of community at work is negatively associated as expected with all technostress creators at both points of time except for techno-uncertainty in T1. Family support is negatively related to techno-invasion, techno-overload, techno-complexity, and techno-insecurity, but only in T2. For the significant relationships, most effect sizes can be regarded as small effects. Exceptions are the effect of co-worker support on techno-insecurity in T2, which is medium, and the effects of supervisor support and the effect of sense of community on techno-uncertainty in T2 as well as the effects of family support on techno-overload and techno-insecurity in T2, which are less than small (Cohen 1988).

To sum up, supervisor support and co-worker support are associated only with some of the technostress creators and the relation is positive and not negative as expected. However, sense of community as well as family support could be effective measures to inhibit technostress creators as they are negatively related to technostress creators. Thus, we can partially support our first hypothesis.

### Comparison of Relationships Between T1 and T2

For the last step of our analysis, we tested whether there are significant changes in relationships between the two points of time in order to test our second hypothesis. Therefore, we conducted clustered regression analyses (accounting for repeated measures for each survey participant) of the interaction of each social support dimension with a binary time variable (T1 = 0, T2 = 1) on each technostress creator. We used factor scores from the prior SEM for the regression analysis. Table 9 presents the results. For purpose of readability, we only include the results for the interactions. The results for the direct effects can be seen in Appendix C.

**Table 9:** Results of the interaction analyses

Relationship	Clustered std. error	Estimate	*p*-value	sig
Supervisor support x time → Techno-invasion	0.055	− 0.011	0.847	
Supervisor support x time → Techno-overload	0.065	− 0.003	0.968	
Supervisor support x time → Techno-complexity	0.061	− 0.014	0.817	
Supervisor support x time → Techno-insecurity	0.053	− 0.097	0.067	
Supervisor support x time → Techno-uncertainty	0.064	− 0.111	0.081	
Co-worker support x time → Techno-invasion	0.067	0.160	0.016	*
Co-worker support x time → Techno-overload	0.074	0.142	0.055	
Co-worker support x time → Techno-complexity	0.066	0.069	0.295	
Co-worker support x time → Techno-insecurity	0.062	0.209	0.001	***
Co-worker support x time → Techno-uncertainty	0.071	0.144	0.044	*
Sense of community at work x time → Techno-invasion	0.080	0.091	0.257	
Sense of community at work x time → Techno-overload	0.092	− 0.028	0.759	
Sense of community at work x time → Techno-complexity	0.080	0.063	0.434	
Sense of community at work x time → Techno-insecurity	0.080	− 0.108	0.178	
Sense of community at work x time → Techno-uncertainty	0.089	− 0.029	0.747	
Family support x time → Techno-invasion	0.066	− 0.250	0.000	***
Family support x time → Techno-overload	0.073	− 0.182	0.012	*
Family support x time → Techno-complexity	0.063	− 0.133	0.035	*
Family support x time → Techno-insecurity	0.061	− 0.160	0.009	**
Family support x time → Techno-uncertainty	0.067	− 0.193	0.004	**

***p < 0.001, **p < 0.01, *p < 0.05

Again, the results show differences between the social support dimensions. Supervisor support as well as sense of community did not significantly change. The relationship between co-worker support with techno-invasion, techno-insecurity, and techno-uncertainty had become more positive in T2. For family support, the effect had become more negative on all five technostress creators. Thus, family support has significantly become more important as technostress inhibitor in T2 in comparison to T1. This is in line with H2. Thus, we also find partial support for our second hypothesis.

## Discussion

The presented research was motivated in two ways: First, technostress research has increasingly investigated possible mitigation of technostress via individual coping or organizational mechanisms. However, social support as technostress inhibitor has been mostly neglected so far even though it is an inhibitor of stress in general and our results show that it is also an inhibitor of technostress. Second, the COVID-19 pandemic has changed the working environment for many employees by increasing their amount of telework, but also for employees who continued to work in the organizations’ offices. In this changed environment, organizational measures such as technical support may be not as effective as during times of strong physical presence in the organizational offices and, thus, individuals had to find other sources of support.

Our results shed light on the effect of social support to inhibit technostress. Thereby, it is important to note that the amount of supervisor support, co-worker support as well as sense of community at work did not significantly change before and during the pandemic. Thus, the results are not influenced by the availability of each source of support but may be due to other conditions which changed during the pandemic. Family support, however, has been slightly less available during the pandemic.

We find that a sense of community at work can be an effective technostress inhibitor and is negatively associated with technostress creators before and during the pandemic. This beneficial effect of sense of community at work is in line with findings on work stress (Cicognani et al. 2009; Gascón et al. 2021) and adds to these prior findings as we found a direct relation with technostress creators rather than with the outcomes of stress. Even though the individual effects are small in size, they may accumulate to a larger effect on technostress overall. Also, small effects may accumulate over time and thereby still be of practical significance (Funder and Ozer 2019).

Supervisor support, in contrast, cannot be confirmed as technostress inhibitor as it is even positively associated with techno-invasion, techno-insecurity, and techno-uncertainty, even though only showing a (very) small effect size. This is in contrast to prior findings on the effect of supervisor support on work stress (e.g., Sass et al. 2011; Sosik and Godshalk 2000). A reason for this may be that supervisors who engage in actively mentoring their employees may decrease the employees’ stress (Sosik and Godshalk 2000). The conceptualization of supervisor support in our study was more to capture whether supervisors care about the employees’ well-being (Eisenberger et al. 2002) but without pro-actively promoting their work activities and abilities. For techno-invasion, the reason for the positive relationship to supervisor support might be that if an employee has a close relationship with his or her supervisor, they are more willing to be contacted during non-work hours when this appears important to the supervisor. For techno-insecurity and techno-uncertainty, the explanation for this positive relationship is less intuitive and needs further investigation.

The same is the case for the discovered positive relationship between co-worker support and techno-insecurity during the pandemic. Apart from that relationship, co-worker support could not be confirmed as technostress inhibitor. Other studies on co-worker support found negative relations to outcomes of stress, such as job dissatisfaction and strain (McCarty et al. 2007; Wolgast and Fischer 2017). This could imply that employees only make use of the support of their co-workers when they already experience technostress, that is, they use it as a coping strategy. This would be in line with Weinert et al. (2020) who found that emotional support from co-workers is an effective coping strategy for situational technostress. However, the effect of co-worker support in the (techno)stress process needs further investigation.

Family support did become slightly lower during the pandemic. Yet, in this whole period of time, it was important as a technostress inhibitor. While it did not have an effect before the pandemic, it significantly decreased technostress creators during the pandemic. Still, the effect size was rather small. However, this is an important finding indicating that employees found alternative sources of support during the pandemic and found this source in their own family members. The findings on family support fall in the realm of boundary spill-over mechanisms between the private and the work domain and thereby add to the findings of Benlian (2020). Another interesting aspect for future research could be to investigate whether social support also has beneficial effects on experiencing challenge technostress rather than hindrance technostress.

For all relationships between social support dimensions and technostress creators in T1 or T2 that were found to be significant, the effect sizes were mostly small with some exceptions being less than small and one exception being medium-sized. However, such small effects can still be important when they accumulate to larger effects (Funder and Ozer 2019). Also, as pointed out by Mohajeri et al. (2020), such findings in social sciences, even though small in effect size, also have to be regarded in terms of their relevance, statistical significance, and practical significance. We already showed statistical significance in Sect. 4 and pointed out the relevance of the finding especially in times of high telework. Also, our results are of practical significance since inhibiting technostress creators may help organizations mitigate negative outcomes of technostress such as reduced employee commitment or productivity.

### Theoretical Contribution

Our results contribute to literature in several ways: First, we extend literature on technostress inhibitors and transfer knowledge from psychology research to the technostress domain. We find that a sense of community at work may help to inhibit technostress creators and that family support has the same effect in work settings with large amounts of telework. This adds to the previously mainly investigated technostress inhibitors (literacy facilitation, involvement facilitation, and technical support provision) and may inspire research to further study the effects of social support on technostress creators and the relationship between technostress creators and strain. For future research, it is important to look into whether different groups of employees (e.g., male vs. female employees) lean on different dimensions of social support. Also, analogously to research on technostress inhibitors, it could be worth investigating whether social support dimensions also have an effect on technostress-induced strain, and if so of which size, or if they show an interaction effect between technostress creators and strain.

Second, we find evidence that not all social support dimensions are related to technostress creators in the same direction. According to our results, supervisor support does not function as technostress inhibitor. Rather, it is positively related with technostress. This is an important finding and shows that social support dimensions have to be differentiated. Future research should analyze the reasons for the differences between different social support dimensions.

Third, we find differing results between the technostress creators. Prior research has often built a higher-order construct of technostress creators (e.g., Ragu-Nathan et al. 2008) instead of investigating the relationship of the first-order constructs with, for example, antecedents and outcomes of technostress. We show the importance of differentiating the various technostress creators.

### Practical Implications

Our results suggest different practical implications for organizations. First, the paper shows that not only organizational mechanisms such as the provision of technical support or training with digital technologies can inhibit technostress but also softer mechanisms such as the sense of community at work. On the one hand, this offers more opportunities for introducing mitigation measures for technostress. For example, team building events to foster a sense of community could then also help to inhibit technostress. On the other hand, however, building such a sense of community among the employees takes time, and it possibly needs several trust- or teambuilding measures rather than just one.

Second, the paper finds evidence that there can be positive spill-over effects from the private to the work domain as family support can help inhibit technostress creators. However, organizations should not rely on this effect to come automatically from the private domain. Rather, they should engage in work-home boundary management programs (such as policies for when to be available or the possibility to shape work time as flexible as needed to manage both the work and the private domain) as also suggested by Benlian (2020) to support employees in managing their boundaries so that positive spill-overs can be realized.

Third, it is important for organizations as well as supervisors to realize that social support is not only beneficial for inhibiting technostress creators since supervisor support was positively associated with some of the technostress creators. Thus, supervisors should avoid their behavior implying too high expectations in terms of, for example, availability during non-working hours when they have a good relationship. According to our results, such a behavior may increase techno-invasion. To overcome this issue, supervisors could challenge their behavior in that regard and actively communicate their expectations on when employees should be available and when not.

Last, we find differing results between the technostress creators. Thus, organizations should change their technostress mitigation efforts from using global measures to mitigate technostress in general and should instead differentiate which measures would help to mitigate which technostress creator. Therefore, they should measure the level of technostress creators to have evidence about which technostress creators are most problematic among their employees and then introduce the matching mitigation measures.

### Limitations

Our study has several limitations. For answering the first hypothesis, we used data from two cross-sectional surveys, which limits the possibility to find causal effects between social support and technostress creators. Even though the causal motivation for each relationship stems from theory and prior literature, future research should follow up with generating further data sets to test robustness and generalizability. Further, the COVID-19 pandemic has come along with a large variety of changes in the private and business environment of employees. Thus, it may be that the surveyed constructs in our study do not completely cover all these changes. Also, we did not control for possible adaptations that individuals may already have made to the changed environment (e.g., whether they already increased their technical competence or found habits of social interaction at the time of T2). Future research should further investigate these changes and adaptations that have not been examined in our study. Further research on social support in a predominantly telework setting is also important in order to draw conclusions for the new normal of working after the COVID-19 pandemic. Repeating the study at one point of time after the pandemic could be a promising path towards this.

## Conclusion

Digitalization as well as the COVID-19 pandemic have dramatically changed workplaces and working environments. The resulting technostress can be inhibited by different organizational mechanisms as well as by support from an individual’s environment. Our results give evidence that social support can be an effective technostress inhibitor and that it becomes even more important when the amount of telework is large. Even when the social distancing measures of the COVID-19 pandemic are terminated, the new normal of working will include greater amounts of telework than before the COVID-19 pandemic as many studies show. Thus, our results remain relevant even after the pandemic and may inspire research and organizations when preparing for the new normal of working.

## Supplementary Information

Below is the link to the electronic supplementary material.

Supplementary file1 (PDF 93 KB)
