# Supplementary material for: Social Support as Technostress Inhibitor: Even More Important During the COVID-19 Pandemic?
Source: Bus Inf Syst Eng. 2023 Mar 21:1–15. Online ahead of print. doi: 10.1007/s12599-023-00799-7 (PMC10029776; doi:10.1007/s12599-023-00799-7)
Supplement: Supplementary file 1 — Supplementary file1 (PDF 93 KB) [file 12599_2023_799_MOESM1_ESM.pdf]

# Social Support as Technostress Inhibitor –
# Even More Important During the COVID-19 Pandemic?

**Julia Lanzl**

Business & Information Systems Engineering (2023)

**Appendix (available online via http://link.springer.com)**

# Appendix A – Measurement Items

| | |
|---|---|
| **Techno-Invasion** (source: Ragu-Nathan et al. 2008)[1)] | |
| TIV01 | I have to be in touch with my work even during my vacation due to this technology. |
| TIV02 | I have to sacrifice my vacation and weekend time to keep current on new technologies. |
| TIV03 | I feel my personal life is being invaded by this technology. |
| **Techno-Overload** (source: Ragu-Nathan et al. 2008)[1)] | |
| TO01 | I am forced to change my work habits to adapt to new technologies. |
| TO02 | I am forced by this technology to work with very tight time schedules. |
| TO03 | I am forced to change my work habits to adapt to new technologies. |
| TO04 | I have a higher workload because of increased technology complexity. |
| **Techno-Complexity** (source: Ragu-Nathan et al. 2008)[1)] | |
| TC01 | I do not know enough about this technology to handle my job satisfactorily. |
| TC02 | I need a long time to understand and use new technologies. |
| TC03 | I do not find enough time to study and upgrade my technology skills. |
| TC04 | I find new recruits to this organization know more about computer technology than I do. |
| TC05 | I often find it too complex for me to understand and use new technologies. |
| **Techno-Insecurity** (source: Ragu-Nathan et al. 2008)[1)] | |
| TIS01 | I feel constant threat to my job security due to new technologies. |
| TIS02 | I have to constantly update my skills to avoid being replaced. |
| TIS03 | I am threatened by coworkers with newer technology skills. |
| TIS04 | I do not share my knowledge with my coworkers for fear of being replaced. |
| TIS05 | I feel there is less sharing of knowledge among coworkers for fear of being replaced. |
| **Techno-Uncertainty** (source: Ragu-Nathan et al. 2008) [1)] | |
| TUC01 | There are always new developments in the technologies we use in our organization. |
| TUC02 | There are constant changes in computer software in our organization. |
| TUC03 | There are constant changes in computer hardware in our organization. |
| TUC04 | There are frequent upgrades in computer networks in our organization. |
| **Supervisor Support** (source: Graen and Uhl-Bien 1995; Schyns 2002)[1)] | |
| SUS01 | My leader understands my job problems and needs. |
| SUS02 | My leader recognizes my potential. |
| SUS03 | My leader would use his/her power to help me solve problems in my work. |
| SUS04 | I have enough confidence in my leader that I would defend and justify his/her decision. |
| SUS05 | Regardless of the amount of formal authority my leader has, he/she would “bail me out”, at his/her expenses. |
| SUS06 | I know how my leader generally assesses me. |
| **Co-Worker Support** (source: Burr et al. 2019)[2)] | |
| SSW01 | How often do you get help and support from your colleagues if needed? |
| SSW02 | How often are your colleagues willing to listen to your problems at work if needed? |
| **Sense of Community at Work** (source: Burr et al. 2019)[2)] | |
| SCW01 | Is there a good atmosphere between you and your colleagues? |
| SCW02 | Do you feel part of a community at your place of work? |

| **Family Support** (source: Graen and Uhl-Bien 1995; Schyns 2002)[1] | |
|---|---|
| FS01 | People from my close private environment (e.g., partner, children, parents) understand my job problems and needs. |
| FS02 | People from my close private environment (e.g., partner, children, parents) would use their possibilities to help me solve problems in my work. |
| FS03 | People from my close private environment (e.g., partner, children, parents) would “bail me out”, at their expenses. |
| FS04 | People from my close private environment (e.g., partner, children, parents) understand my private problems and needs. |
| FS05 | I know how people from my close private environment (e.g., partner, children, parents) generally assess me. |

[1] Measured on a five-point Likert scale ranging from “strongly disagree” to “strongly agree”.
[2] Measured on a six-point Likert scale ranging from “never” to “always”.

# Appendix B – Fornell-Larcker Criterion

## Inter-Factor-Correlations for T1 (square root of AVE in the diagonal)

| | TIV | TO | TC | TIS | TUC | SUS | CWS | SCW | FS |
|---|---|---|---|---|---|---|---|---|---|
| Techno-Invasion (TIV) | 0.782 | | | | | | | | |
| Techno-Overload (TO) | 0.577 | 0.832 | | | | | | | |
| Techno-Complexity (TC) | 0.608 | 0.626 | 0.825 | | | | | | |
| Techno-Insecurity (TIS) | 0.687 | 0.720 | 0.621 | 0.762 | | | | | |
| Techno-Uncertainty (TUC) | 0.473 | 0.592 | 0.477 | 0.659 | 0.800 | | | | |
| Supervisor Support (SUS) | -0.020 | -0.133 | -0.130 | -0.021 | 0.094 | 0.840 | | | |
| Co-Worker Support (CWS) | -0.244 | -0.190 | -0.182 | -0.233 | -0.057 | 0.282 | 0.825 | | |
| Sense of Community (SCW) | -0.369 | -0.303 | -0.343 | -0.311 | -0.078 | 0.343 | 0.525 | 0.905 | |
| Family Support (FS) | -0.035 | -0.070 | -0.125 | -0.070 | 0.081 | 0.307 | 0.306 | 0.275 | 0.777 |

## Inter-Factor-Correlations for T2 (square root of AVE in the diagonal)

| | TIV | TO | TC | TIS | TUC | SUS | CWS | SCW | FS |
|---|---|---|---|---|---|---|---|---|---|
| Techno-Invasion (TIV) | 0.778 | | | | | | | | |
| Techno-Overload (TO) | 0.654 | 0.854 | | | | | | | |
| Techno-Complexity (TC) | 0.634 | 0.665 | 0.839 | | | | | | |
| Techno-Insecurity (TIS) | 0.767 | 0.756 | 0.711 | 0.786 | | | | | |
| Techno-Uncertainty (TUC) | 0.471 | 0.588 | 0.508 | 0.626 | 0.847 | | | | |
| Supervisor Support (SUS) | -0.064 | -0.187 | -0.173 | -0.059 | -0.201 | 0.852 | | | |
| Co-Worker Support (CWS) | -0.107 | -0.152 | -0.161 | -0.007 | -0.171 | 0.538 | 0.849 | | |
| Sense of Community (SCW) | -0.273 | -0.315 | -0.307 | -0.106 | -0.397 | 0.475 | 0.634 | 0.912 | |
| Family Support (FS) | -0.268 | -0.224 | -0.260 | -0.112 | -0.272 | 0.380 | 0.337 | 0.380 | 0.804 |

## Appendix C – Results for Direct Effects of the Regression Analysis

| Relationship | Clustered Std. Error | Estimate | p-value | sig. |
|---|---|---|---|---|
| Supervisor Support → Techno-Invasion | 0.044 | 0.095 | 0.030 | * |
| Supervisor Support → Techno-Overload | 0.053 | -0.047 | 0.376 | |
| Supervisor Support → Techno-Complexity | 0.048 | -0.007 | 0.881 | |
| Supervisor Support → Techno-Insecurity | 0.044 | 0.078 | 0.072 | |
| Supervisor Support → Techno-Uncertainty | 0.045 | 0.094 | 0.037 | * |
| Co-Worker Support → Techno-Invasion | 0.052 | -0.091 | 0.080 | |
| Co-Worker Support → Techno-Overload | 0.058 | -0.053 | 0.357 | |
| Co-Worker Support → Techno-Complexity | 0.052 | -0.014 | 0.781 | |
| Co-Worker Support → Techno-Insecurity | 0.051 | -0.114 | 0.024 | * |
| Co-Worker Support → Techno-Uncertainty | 0.055 | -0.061 | 0.263 | |
| Sense of Community at Work → Techno-Invasion | 0.061 | -0.393 | 0.000 | *** |
| Sense of Community at Work → Techno-Overload | 0.061 | -0.327 | 0.000 | *** |
| Sense of Community at Work → Techno-Complexity | 0.058 | -0.368 | 0.000 | *** |
| Sense of Community at Work → Techno-Insecurity | 0.061 | -0.310 | 0.000 | *** |
| Sense of Community at Work → Techno-Uncertainty | 0.060 | -0.122 | 0.041 | * |
| Family Support → Techno-Invasion | 0.052 | 0.023 | 0.657 | |
| Family Support → Techno-Overload | 0.058 | 0.032 | 0.589 | |
| Family Support → Techno-Complexity | 0.051 | -0.052 | 0.303 | |
| Family Support → Techno-Insecurity | 0.050 | 0.005 | 0.928 | |
| Family Support → Techno-Uncertainty | 0.053 | 0.088 | 0.098 | |
| Time → Techno-Invasion | 0.228 | 0.177 | 0.438 | |
| Time → Techno-Overload | 0.262 | 0.198 | 0.449 | |
| Time → Techno-Complexity | 0.239 | 0.098 | 0.681 | |
| Time → Techno-Insecurity | 0.234 | 0.408 | 0.082 | |
| Time → Techno-Uncertainty | 0.249 | 0.298 | 0.231 | |

Note: *** $p < 0.001$, ** $p < 0.01$, * $p < 0.05$
